# Acute psychological responses to formal and small-sided volleyball games in male youth athletes: an exploratory repeated-measures study

**DOI:** 10.3389/fpsyg.2026.1832942

**Published:** 2026-06-17

**Authors:** Marcos Henrique do Nascimento, Alberto Souza de Sá Filho, Vicente Aprigliano, Filipe Manuel Clemente, Carlos Alexandre Vieira, Mário Hebling Campos, Augusto Cezar Rodrigues Rocha, Débora Darck Lopes Costa Arantes, Gibson Moreira Praça, Auro Barreiros Freire, Adam Kawczyński, Matias Noll, Gustavo De Conti Teixeira Costa

**Affiliations:** 1 Faculdade de Educação Física, Universidade Federal de Goiás, Goiânia, Brazil; 2 Universidade Evangélica de Goiás, Anápolis, Brazil; 3 Escuela de Ingeniería de Construcción y Transporte, Pontificia Universidad Católica de Valparaíso, Valparaíso, Chile; 4 Gdansk University of Physical Education and Sport, Gdańsk, Poland; 5 Applied Research Institute (i2A), Sport Physical Activity and Health Research and Innovation Center, Polytechnic University of Coimbra, Coimbra, Portugal; 6 Escola de Educação Física, Fisioterapia e Terapia Ocupacional, Universidade Federal de Minas Gerais, Belo Horizonte, Brazil; 7 Instituto Federal de Minas Gerais, Campus Sabará, Sabará, Brazil; 8 Faculty of Medicine, Wrocław University of Science and Technology, Wrocław, Poland; 9 Instituto Federal de Educação, Ciência e Tecnologia Goiano, Ceres, Brazil

**Keywords:** enjoyment, mood state, small-sided games, state anxiety, volleyball, youth athletes

## Abstract

**Introduction:**

Small-sided games are frequently used in youth sport training, yet their acute psychological effects in volleyball remain insufficiently described. This study examined whether formal games (6 vs. 6) and small-sided games (3 vs. 3) were associated with distinct short-term responses in anxiety, mood, affective valence, enjoyment, perceived recovery, and perceived exertion among male youth volleyball athletes.

**Methods:**

Thirty male athletes aged 14–18 years completed repeated training sessions under two game formats during the pre-competitive period. Psychological and perceptual measures were obtained before and/or after each session, depending on the outcome assessed. Generalized Estimating Equations were used to examine condition, moment, and condition × moment effects. Given the sample size, the nonrandomized repeated-measures design, and the fixed sequence of conditions, the findings should be interpreted as exploratory, as condition effects cannot be fully separated from order and time-related influences.

**Results:**

State anxiety increased from pre- to post-session, irrespective of game format. Among the mood-related outcomes, only tension demonstrated a statistically robust condition × moment interaction after false discovery rate correction, with less favorable post-session responses in the 3 vs. 3 format. Depression, fatigue, and confusion increased, and vigor decreased from pre- to post-session regardless of condition. Affective valence, perceived recovery, and perceived exertion did not differ between formats. Although anger and enjoyment showed condition-related differences in the unadjusted analyses, these findings did not remain statistically robust after correction for multiple comparisons.

**Discussion:**

In this exploratory sample of male youth volleyball athletes, formal and small-sided games produced broadly similar acute psychological responses across most outcomes. The most robust condition-related finding was higher post-session tension in the small-sided format, whereas the unadjusted findings for anger and enjoyment should be interpreted with caution, as they did not remain statistically robust after false discovery rate correction. These findings suggest that different game formats may be associated with specific short-term emotional experiences during training, but larger, better-controlled studies are needed before drawing definitive conclusions about their implications for mental health-related outcomes in youth sport.

## 1 Introduction

Competitive youth sport involves substantial emotional demands, and acute changes in anxiety, mood, affective valence, and enjoyment may shape both the quality of the training experience and athletes’ short-term psychological responses (Carter et al., 2021; Jekauc et al., 2021). In team-sport settings, pre-competitive anxiety and negative mood fluctuations are particularly relevant because they may influence how athletes perceive task demands, regulate emotions, and respond to sport participation (McDonald et al., 2023; Putukian and Yeates, 2023).

Volleyball is a particularly relevant context for examining these responses. The sport combines technical precision, tactical organization, rapid decision-making, and interpersonal coordination, all of which may increase acute cognitive and emotional demands in youth athletes (Millán-Sánchez et al., 2023; de Andrade Nogueira et al., 2024). Previous studies in volleyball have shown that emotional states such as anxiety and mood may fluctuate according to situational and competitive conditions, suggesting that training tasks may not be psychologically neutral for developing players (Rotta et al., 2014; Vavassori et al., 2023).

One methodological strategy frequently used in sport training is the adoption of small-sided games (SSG). By manipulating the number of players, playing area, and task dynamics, SSGs are designed to modify technical-tactical involvement and may also influence psychological responses such as enjoyment, engagement, and perceived demands (Clemente et al., 2021; Clemente et al., 2022). Although SSG research has expanded across team sports, most evidence comes from invasion games, and less is known about their acute psychological implications in net sports such as volleyball, especially in youth categories (Clemente et al., 2021; Fernández-Espínola et al., 2020; Gok et al., 2023).

In volleyball, reduced-player formats may increase individual exposure, ball involvement, and decisional demands, whereas the formal game may better reproduce the tactical organization and role specialization experienced in competition (Nascimento et al., 2023; Rocha et al., 2020). These task-related differences may influence not only technical-tactical engagement, but also the perceptual-cognitive and emotional demands experienced by youth athletes during training. From this perspective, 3 vs. 3 and 6 vs. 6 formats may not be psychologically equivalent, even when performed within the same training context. However, to our knowledge, no study has directly compared the acute psychological responses associated with small-sided and formal volleyball games in male youth athletes.

Accordingly, the aim of this exploratory repeated-measures study was to compare the acute responses of male youth volleyball athletes to a small-sided game format (3 vs. 3) and a formal game format (6 vs. 6). The primary outcomes were state anxiety and mood-state dimensions. Secondary outcomes were affective valence, enjoyment, perceived recovery before sessions, and rating of perceived exertion after sessions. Given the design and sample characteristics, the analyses were approached as exploratory rather than confirmatory, and any observed between-format differences should be interpreted cautiously. Even within this exploratory framework, we expected that the reduced format might be associated with less favorable acute emotional responses due to its potentially greater individual exposure, higher action density, and greater perceptual-cognitive demands.

## 2 Materials and methods

### 2.1 Experimental approach

The reporting of this repeated-measures study adhered to the Transparent Reporting of Evaluations with Nonrandomized Designs (TREND) statement. We employed an exploratory repeated-measures design, and the present study was approved by the Research Ethics Committee under protocol CAAE: 65290217.2.0000.5083. Because the same athletes were repeatedly assessed across multiple sessions under both game formats, the study enabled within-subject comparisons of acute psychological and perceptual responses. However, because the sequence of conditions was fixed rather than randomized or counterbalanced, the design should be interpreted as a repeated-measures, exploratory design rather than a classical crossover trial.

Because all participants followed the same fixed sequence of conditions across sessions, potential effects of session order, repeated exposure, temporal progression, and pre-competitive contextual variation may have confounded the interpretation of condition-related differences. Consequently, the present design does not allow a clear separation between format-related effects and time-related influences, thereby limiting causal inference regarding the acute psychological responses associated with each game format. Although repeated exposure to each format may have reduced part of the intra-individual variability associated with isolated sessions, these procedures do not eliminate potential order bias. Therefore, the findings should be interpreted as exploratory and hypothesis-generating. Future studies should adopt randomized, counterbalanced, crossover, repeated-measures designs to better isolate the specific effects of game format.

### 2.2 Participants

Thirty male volleyball athletes aged 14–18 years (M = 16.2; SD = 1.1) participated. They represented youth-category teams linked to state development projects in Minas Gerais, were state championship finalists, and ranked among the top four teams at the Brazilian interclub championship. All were enrolled in school and engaged in systematic training (≥4 sessions/week). Inclusion criteria were ≥2 years of competitive volleyball experience, no injuries/conditions precluding training at data collection, and parental/guardian consent. Exclusion criteria were failure to complete questionnaires on any collection day, absence on any intervention day, or >3 missed training sessions in the 15 days prior to testing. No sample attrition occurred, and no cases were excluded after eligibility screening.

Recruitment was therefore determined by the number of eligible athletes available within the competitive team context in which the study was conducted. Accordingly, the sample should be understood as a convenience sample embedded in a repeated-measures design, which increases the number of observations per participant and supports within-subject comparisons, but does not eliminate the need for larger samples in future confirmatory studies. For this reason, the present findings should be interpreted as exploratory and hypothesis-generating rather than as providing definitive evidence regarding between-format differences.

All participants and their legal guardians were informed about the study and, prior to its initiation, read and signed the Informed Consent Form and the Informed Assent Form. Additionally, the study adhered to the principles of the 1964 Declaration of Helsinki and its subsequent amendments, or to comparable ethical standards. Finally, data were anonymized before analysis by removing direct identifiers (e.g., names, contact details), and only authorized investigators had access to the analytical dataset.

### 2.3 Design and interventions

Participants completed two volleyball training formats on separate days: a small-sided game condition (3 vs. 3) and a formal game condition (6 vs. 6). Each format was performed in three experimental sessions, following one prior familiarization session with the instruments and data-collection routine. Each experimental session lasted 33 min and consisted of four 6-min sets interspersed with 3 min of passive recovery. A standardized warm-up was performed before every session and included passive stretching, locomotor exercises, and ball-based activities. The 3 vs. 3 condition was played on a reduced-size court (18 × 4.5 m), whereas the 6 vs. 6 condition followed the official format on a full court (18 × 9.0 m). Official volleyball rules were adopted in both conditions. Sessions were conducted at the same time of day and under similar environmental conditions.

Anxiety, mood, and affective valence were assessed before and immediately after each session. Perceived recovery was measured before each session, while enjoyment and rating of perceived exertion were measured after each session. All instruments were self-administered digitally in a quiet area adjacent to the court, following standardized instructions. Because the order of experimental conditions was fixed across sessions, order and time-related effects (e.g., learning, habituation, fatigue accumulation, or changes in psychological state across the pre-competitive period) cannot be separated from condition effects. Consequently, any observed differences between formats should not be interpreted solely as attributable to the game structure.

All sessions were supervised by the same coaching staff, who provided standardized verbal instructions focused on maintaining game flow and adherence to official rules, without systematic motivational feedback or manipulation of encouragement. Players were rotated between sets to ensure similar participation time for each athlete within each session. Multiple balls were available around the court to minimize interruptions and maintain task continuity. No additional task constraints (e.g., rule modifications, scoring adaptations, or role restrictions) were introduced beyond those inherent to each format (3 vs. 3 vs. 6 vs. 6). These procedures were adopted to standardize the training environment and improve reproducibility of the intervention.

### 2.4 Outcome measures

**Figure 1:**
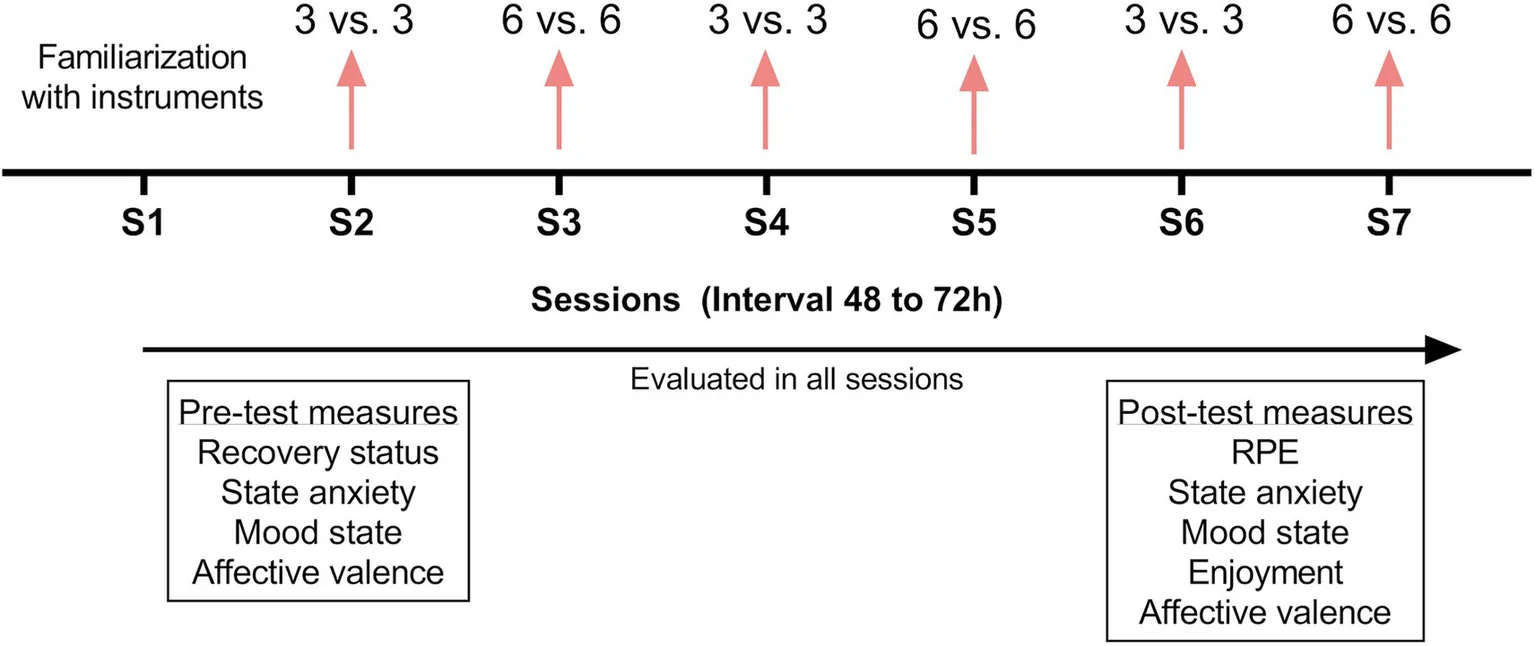
Experimental design.

Psychological and perceptual outcomes were assessed using validated instruments widely applied in sport and exercise psychology research. Data collection took place during the pre-competition period, approximately 20 days before the start of the competition, during regular training hours (4:00–6:00 p.m.) to control for circadian influences. In the week preceding the intervention, athletes completed a familiarization session with all questionnaires to minimize misunderstanding and response bias. During the experimental period, the instruments were always self-administered on a digital platform, individually, on the training court, in a quiet area adjacent to the playing area. In each experimental session, the questionnaires were administered 20 min before the beginning of the intervention and immediately after its completion. The researcher distributed the questionnaires, provided standardized instructions, and remained available to clarify doubts, while the coaching staff supervised the entire procedure. The instruments were administered in the same fixed order in all sessions, following the sequence depicted in Figure 1.

#### 2.4.1 Variables analyzed

State anxiety: State anxiety was measured with the Brazilian version of the State–Trait Anxiety Inventory (IDATE), State subscale (Biaggio and Natalício, 1979), developed initially by Spielberg (1971), translated and adapted to Brazilian Portuguese following standardized procedures (Biaggio et al., 1977). Brazilian studies report adequate factorial structure and internal consistency for the IDATE across different populations, supporting its construct validity and reliability (Andrade et al., 2001; Biaggio, 2018; Kaipper et al., 2010).

Mood state: was assessed using the Brazilian 24-item version of the Brunel Mood Scale (BRUMS), derived from the Profile of Mood States (POMS) and adapted for athletes and physically active individuals. The Brazilian BRUMS underwent translation, back translation, and psychometric testing in Brazilian samples, demonstrating satisfactory validity and reliability for detecting altered mood states in athletes (Rohlfs et al., 2004; Rohlfs et al., 2008).

Affective valence: was assessed using the Feeling Scale (Hardy and Rejeski, 1989), an 11-point bipolar measure ranging from −5 (“very bad”) to +5 (“very good”), adapted and tested for reproducibility in Brazilian Portuguese, with acceptable measurement properties for use in exercise contexts (Alves et al., 2019).

Enjoyment: was quantified post-session through the Physical Activity Enjoyment Scale (PACES) (Moore et al., 2009), which reflects the extent to which the training experience was perceived as enjoyable and intrinsically rewarding. PACES has been translated and culturally adapted, and has demonstrated satisfactory reliability and reproducibility in Brazilian samples engaged in physical activity (Alves et al., 2019; Monteiro et al., 2017).

Subjective perception of effort (RPE): was recorded immediately after each session using the Borg Rating of Perceived Exertion scale (CR-10 format). The Borg scales are extensively validated for monitoring exercise intensity, including in Portuguese-speaking and Brazilian populations, with studies reporting appropriate validity and reliability indices and recommending their use for quantifying internal load in exercise and sport settings (Camargo et al., 2019; Flôres et al., 2025).

Perceived recovery (TQR): status was measured before each session using the Total Quality Recovery scale, initially proposed by (Kenttä and Hassmén, 1998) as a 6–20 point scale structurally analogous to Borg’s RPE, in which higher scores indicate better overall recovery and subsequently investigated in Brazilian athletes (Osiecki et al., 2015). Studies suggest that TQR is a practical psychometric tool, sensitive to variations in training load and helpful in monitoring recovery state in sports (Brandão et al., 2019; Nogueira et al., 2015).

All psychological and perceptual variables were collected in each experimental session for both conditions (3 vs. 3 and 6 vs. 6), resulting in a repeated-measures structure for each athlete across six training sessions. This design allowed for within-subject comparisons between small-sided and formal games, although the fixed sequence of sessions should be considered when interpreting the findings. Sessions were separated by 48–72 h and conducted under similar environmental and scheduling conditions to reduce residual fatigue and potential carryover or learning effects. These procedures were intended to reduce contextual variability across sessions; however, they do not eliminate potential order effects or fully protect internal validity given the fixed sequence of conditions.

### 2.5 Data analysis

Data were analyzed using Generalized Estimating Equations (GEE), considering their suitability for repeated-measures designs, correlated observations, small samples, and outcomes that may deviate from normality (de Melo et al., 2022). The repeated-measures structure reflected observations nested within each participant across sessions, conditions, and, when applicable, assessment moments. For variables assessed before and after each session, the repeated observations were organized according to session, condition, and moment. For variables assessed only once per session, the repeated observations were organized according to session and condition.

In the GEE models, participants were treated as subjects to account for within-subject dependence across repeated observations. For outcomes assessed before and after the sessions, condition, moment, and the condition × moment interaction were entered as fixed effects. For outcomes assessed only once per session, namely enjoyment, perceived recovery, and rating of perceived exertion, condition was entered as the fixed effect. The session variable was used to define the repeated-measures structure within each participant, but it was not included as an explicit fixed effect in the models. Therefore, the analyses did not formally test session-level time trends, adaptation across repeated exposures, or order-related effects as model predictors. When significant main effects or interactions were identified, pairwise comparisons were explored using Bonferroni-adjusted *post hoc* tests.

The model specification was defined based on the measurement characteristics and empirical distributions of each dependent variable. State anxiety, mood-state dimensions assessed by the BRUMS, affective valence, enjoyment, perceived recovery, and rating of perceived exertion were treated as continuous outcomes and analyzed using GEE models with a normal distribution and an identity link function. Working correlation structures were examined during model specification, and the final models retained an independent working correlation structure to account for within-subject correlation across repeated observations. This choice was based on model stability, convergence behavior, and the relatively small sample size, which may limit the reliability of more complex correlation structures. Robust (sandwich) standard errors were used in all models to provide consistent parameter estimates even under possible misspecification of the working correlation structure. Model fit was assessed using the Quasi-likelihood under the Independence Model Criterion, with lower values indicating better fit.

In addition to the GEE-based analyses, Cohen’s d effect sizes were calculated separately from the raw observed data for the main comparisons in order to provide a standardized measure of magnitude. These effect sizes were interpreted as trivial (<0.20), small (0.20–0.60), moderate (0.61–1.20), large (1.21–2.00), or very large (>2.00) (Fritz et al., 2012). Accordingly, the effect-size estimates should be interpreted as complementary descriptive indicators and not as parameters derived directly from the GEE models.

Because multiple outcomes and model effects were examined, the analyses were interpreted within an exploratory framework. To reduce the risk of Type I error, false discovery rate correction using the Benjamini–Hochberg procedure was applied to the primary GEE model-effect tests, namely the condition × moment interaction for outcomes assessed before and after the sessions and the condition effect for outcomes assessed once per session. Unadjusted *p*-values are reported to preserve transparency, whereas the FDR-adjusted interpretation was used to qualify the robustness of the findings. Statistical significance was set at *p* ≤ 0.05 for unadjusted analyses and q ≤ 0.05 for FDR-adjusted interpretation. All analyses were performed in IBM SPSS Statistics for Windows, version 26.0 (IBM Corp., Armonk, NY, USA).

## 3 Results

The descriptive values in Table 1 represent pooled observations across all sessions for each condition, expressed as means and standard deviations of session-level data aggregated at the group level. Therefore, these values do not correspond to a single measurement point but reflect the overall distribution of repeated observations across the experimental period. For state anxiety, the GEE model was fitted with an independent working correlation structure (QIC_State anxiety = 15095.639). In model effects tests, there was a main effect of moment (Wald *χ*^2^(1) = 10.606; *p* = 0.001), whereas neither the main effect of condition (Wald *χ*^2^(1) = 0.043; *p* = 0.836) nor the condition × moment interaction (Wald *χ*^2^(1) = 0.001; *p* = 0.976) reached significance. Estimated marginal means indicated no difference between formats (3 vs. 3: M = 40.711; SE = 0.628; 95% CI [39.481; 41.941]; 6 vs. 6: M = 40.850; SE = 0.809; 95% CI [39.265; 42.435]; *Δ* = −0.139; SE = 0.671; *p* = 0.836; 95% CI [−1.454; 1.177]). In contrast, state anxiety was higher at post-test than at pre-test (Post: M = 41.761; SE = 0.838; 95% CI [40.118; 43.404]; Pre: M = 39.800; SE = 0.549; 95% CI [38.724; 40.876]; *Δ* = 1.961; SE = 0.602; *p* = 0.001; 95% CI [0.781; 3.141]; *d* = 0.50), indicating a small effect.

**Table 1:** Descriptive data.

		Condition 3 vs. 3		Condition 6 vs. 6	
		Average ± SD		Average ± SD	
		Pre	Post	Pre	Post
State Anxiety (a.u.)		39.72 ± 5.09	41.70 ± 7.36	39.88 ± 5.88	41.82 ± 7.40
Mood State (a.u.)	Tension	2.62 ± 1.93	3.38 ± 3.43	2.76 ± 2.00	2.02 ± 2.20
	Depression	1.09 ± 2.34	2.63 ± 3.93	1.38 ± 2.70	2.22 ± 4.38
	Anger	1.12 ± 2.45	3.36 ± 4.35	1.46 ± 2.96	2.31 ± 4.13
	Vigor	11.97 ± 3.52	10.91 ± 4.03	12.07 ± 3.62	10.66 ± 3.77
	Fatigue	3.72 ± 3.51	4.18 ± 2.94	3.33 ± 3.29	4.38 ± 3.45
	Confusion	0.94 ± 1.49	1.72 ± 3.37	1.02 ± 1.80	1.31 ± 2.48
Affective valence (a.u.)		2.64 ± 1.76	2.70 ± 2.54	2.89 ± 1.84	2.49 ± 2.54
Enjoyment (a.u.)			90.16 ± 25.50		99.11 ± 22.95
TQR (a.u.)		15.29 ± 2.69		15.12 ± 2.67	
RPE (a.u.)			4.26 ± 2.37		4.29 ± 2.35

a.u., unidades arbitrárias.

Mood-state dimensions were analyzed using the GEE specification described in the Methods section. For tension, the GEE model was fitted with an independent working correlation structure (QIC_Tension = 2180.363). In model effects tests, there was a significant condition × moment interaction (Wald *χ*^2^(1) = 7.465; *p* = 0.006), whereas the main effects of condition (Wald *χ*^2^(1) = 3.102; *p* = 0.078) and moment (Wald *χ*^2^(1) = 0.001; *p* = 0.971) were not significant. Estimated marginal means for the interaction showed that, in the 3 vs. 3 condition, tension tended to be higher at post-test than at pre-test (Post: M = 3.378; SE = 0.469; 95% CI [2.458; 4.298]; Pre: M = 2.622; SE = 0.257; 95% CI [2.119; 3.125]; *Δ* = 0.756; SE = 0.488; *p* = 0.122; 95% CI [−0.201; 1.712]). In the 6 vs. 6 condition, the pattern was reversed, with lower tension at post-test than at pre-test (Post: M = 2.022; SE = 0.240; 95% CI [1.551; 2.493]; Pre: M = 2.756; SE = 0.200; 95% CI [2.364; 3.147]; *Δ* = −0.733; SE = 0.308; *p* = 0.017; 95% CI [−1.336; −0.130]). In addition, at post-test the 3 vs. 3 condition showed higher tension than the 6 vs. 6 condition (Δ = 1.356; SE = 0.497; *p* = 0.006; 95% CI [0.381; 2.330]; *d* = 0.65), indicating a small-to-moderate effect, whereas at pre-test no between-condition difference was observed (Δ = −0.133; SE = 0.377; *p* = 0.724; 95% CI [−0.872; 0.606]). Overall, the Bonferroni *post hoc* tests also indicated lower tension scores in the 6 vs. 6 condition than in the 3 vs. 3 condition (*p* = 0.018; *d* = 0.24), corresponding to a small effect.

For depression, the GEE model was fitted with an independent working correlation structure (QIC_Depression = 4227.188). In model effects tests, there was a main effect of moment (Wald *χ*^2^(1) = 10.217; *p* = 0.001), whereas the main effect of condition (Wald *χ*^2^(1) = 0.050; *p* = 0.824) and the condition × moment interaction (Wald *χ*^2^(1) = 1.323; *p* = 0.250) were not significant. Estimated marginal means indicated no difference between formats (3 vs. 3: M = 1.861; SE = 0.303; 95% CI [1.268; 2.454]; 6 vs. 6: M = 1.800; SE = 0.356; 95% CI [1.102; 2.498]; *Δ* = 0.061; SE = 0.274; *p* = 0.824; 95% CI [−0.476; 0.598]). By contrast, depression was higher at post-test than at pre-test (Post: M = 2.428; SE = 0.439; 95% CI [1.568; 3.287]; Pre: M = 1.233; SE = 0.242; 95% CI [0.759; 1.707]; *Δ* = 1.194; SE = 0.374; *p* = 0.001; 95% CI [0.462; 1.927]; *d* = 0.61), indicating a moderate effect.

For anger, the GEE model was fitted with an independent working correlation structure (QIC_Anger = 4523.055). In the unadjusted model-effect tests, there was a main effect of moment (Wald *χ*^2^(1) = 21.685; *p* < 0.001), whereas the main effect of condition was not significant (Wald *χ*^2^(1) = 0.985; *p* = 0.321). The unadjusted condition × moment interaction was also significant (Wald *χ*^2^(1) = 4.794; *p* = 0.029), but this interaction did not remain statistically robust after false discovery rate correction and should therefore be interpreted cautiously as exploratory. Estimated marginal means for the interaction indicated that anger increased from pre- to post-test in the 3 vs. 3 condition (Post: M = 3.356; SE = 0.517; 95% CI [2.342; 4.369]; Pre: M = 1.122; SE = 0.287; 95% CI [0.560; 1.685]; *Δ* = 2.233; SE = 0.548; *p* < 0.001; 95% CI [1.159; 3.307]) and also in the 6 vs. 6 condition (Post: M = 2.311; SE = 0.403; 95% CI [1.521; 3.101]; Pre: M = 1.456; SE = 0.355; 95% CI [0.759; 2.152]; *Δ* = 0.856; SE = 0.392; *p* = 0.029; 95% CI [0.086; 1.625]). In addition, at post-test, anger was higher in the 3 vs. 3 than in the 6 vs. 6 condition in the unadjusted comparison (Δ = 1.044; SE = 0.528; *p* = 0.048; 95% CI [0.010; 2.079]; *d* = 0.40), corresponding to a small effect, whereas at pre-test the between-condition difference was not significant (Δ = −0.333; SE = 0.457; *p* = 0.465; 95% CI [−1.229; 0.562]). Considering the main effect of moment, Bonferroni *post hoc* tests indicated higher anger scores at post-test than at pre-test (*p* = 0.001; *d* = 0.87), corresponding to a moderate effect.

For fatigue, the GEE model was fitted with an independent working correlation structure (QIC_Fatigue = 3900.092). In model effects tests, there was a main effect of moment (Wald *χ*^2^(1) = 8.471; *p* = 0.004), whereas neither the main effect of condition (Wald *χ*^2^(1) = 0.096; *p* = 0.756) nor the condition × moment interaction (Wald *χ*^2^(1) = 0.599; *p* = 0.439) was significant. Estimated marginal means indicated no difference between formats (3 vs. 3: M = 3.950; SE = 0.313; 95% CI [3.337; 4.563]; 6 vs. 6: M = 3.856; SE = 0.385; 95% CI [3.100; 4.611]; *Δ* = 0.094; SE = 0.304; *p* = 0.756; 95% CI [−0.502; 0.691]). Fatigue was higher at post-test than at pre-test (Post: M = 4.278; SE = 0.309; 95% CI [3.672; 4.883]; Pre: M = 3.528; SE = 0.371; 95% CI [2.800; 4.255]; Δ = 0.750; SE = 0.258; *p* = 0.004; 95% CI [0.245; 1.255]; *d* = 0.39), indicating a small effect.

For confusion, the GEE model was fitted with an independent working correlation structure (QIC_Confusion = 2053.760). In model effects tests, there was a main effect of moment (Wald *χ*^2^(1) = 4.055; *p* = 0.044), whereas the main effect of condition (Wald *χ*^2^(1) = 0.342; *p* = 0.559) and the condition × moment interaction (Wald *χ*^2^(1) = 0.842; *p* = 0.359) were not significant. Estimated marginal means showed no difference between formats (3 vs. 3: M = 1.333; SE = 0.198; 95% CI [0.946; 1.721]; 6 vs. 6: M = 1.167; SE = 0.210; 95% CI [0.756; 1.578]; *Δ* = 0.167; SE = 0.285; *p* = 0.559; 95% CI [−0.392; 0.725]). However, confusion was higher at post-test than at pre-test (Post: M = 1.517; SE = 0.250; 95% CI [1.026; 2.007]; Pre: M = 0.983; SE = 0.122; 95% CI [0.744; 1.223]; Δ = 0.533; SE = 0.265; *p* = 0.044; 95% CI [0.014; 1.052]; *d* = 0.49), indicating a small effect.

For vigor, the GEE model was fitted with an independent working correlation structure (QIC_Vigor = 4995.787). In model effects tests, there was a main effect of moment (Wald *χ*^2^(1) = 10.193; *p* = 0.001), whereas neither the main effect of condition (Wald *χ*^2^(1) = 0.036; *p* = 0.849) nor the condition × moment interaction (Wald *χ*^2^(1) = 0.218; *p* = 0.641) was significant. Estimated marginal means indicated no difference between formats (3 vs. 3: M = 11.439; SE = 0.399; 95% CI [10.656; 12.221]; 6 vs. 6: M = 11.361; SE = 0.378; 95% CI [10.621; 12.101]; *Δ* = 0.078; SE = 0.408; *p* = 0.849; 95% CI [−0.723; 0.878]). Vigor was lower at post-test than at pre-test (Post: M = 10.783; SE = 0.407; 95% CI [9.986; 11.581]; Pre: M = 12.017; SE = 0.357; 95% CI [11.316; 12.717]; Δ = −1.233; SE = 0.386; *p* = 0.001; 95% CI [−1.990; −0.476]; *d* = 0.58), indicating a small effect.

For affective valence, the GEE model was fitted with an independent working correlation structure (QIC_Affective valence = 1737.621). In model effects tests, there was no main effect of condition (Wald *χ*^2^(1) = 0.006; *p* = 0.939), no main effect of moment (Wald *χ*^2^(1) = 0.562; *p* = 0.454), and no condition × moment interaction (Wald *χ*^2^(1) = 1.870; *p* = 0.171). Estimated marginal means confirmed the absence of differences between formats (3 vs. 3: M = 2.672; SE = 0.247; 95% CI [2.188; 3.156]; 6 vs. 6: M = 2.689; SE = 0.261; 95% CI [2.178; 3.200]; *Δ* = −0.017; SE = 0.217; *p* = 0.939; 95% CI [−0.443; 0.409]) and between moments (Pre: M = 2.767; SE = 0.224; 95% CI [2.328; 3.205]; Post: M = 2.594; SE = 0.286; 95% CI [2.034; 3.155]; Δ = −0.172; SE = 0.230; *p* = 0.454; 95% CI [−0.623; 0.278]).

For enjoyment, the GEE model was fitted with an independent working correlation structure (QIC_Enjoyment = 104736.111). In the unadjusted model-effect test, there was a main effect of condition (Wald *χ*^2^(1) = 5.922; *p* = 0.015), but this effect did not remain statistically robust after false discovery rate correction and should therefore be interpreted cautiously as exploratory. Estimated marginal means indicated higher enjoyment in the 6 vs. 6 format than in the 3 vs. 3 format in the unadjusted comparison (6 vs. 6: M = 99.111; SE = 2.372; 95% CI [94.462; 103.761]; 3 vs. 3: M = 90.156; SE = 3.439; 95% CI [83.415; 96.896]; *Δ* = 8.956; SE = 3.680; *p* = 0.015; 95% CI [1.742; 16.169]; *d* = 0.54), corresponding to a small effect size.

For perceived recovery, the GEE model was fitted with an independent working correlation structure (QIC_TQR = 1284.995). In model effects tests, there was no main effect of condition (Wald *χ*^2^(1) = 0.232; *p* = 0.630). Estimated marginal means indicated similar recovery scores in the 3 vs. 3 and 6 vs. 6 formats (3 vs. 3: M = 15.289; SE = 0.309; 95% CI [14.682; 15.895]; 6 vs. 6: M = 15.122; SE = 0.310; 95% CI [14.515; 15.729]; *Δ* = 0.167; SE = 0.346; *p* = 0.630; 95% CI [−0.511; 0.845]).

For rating of perceived exertion, the GEE model was fitted with an independent working correlation structure (QIC_RPE = 999.267). In model effects tests, there was no main effect of condition (Wald *χ*^2^(1) = 0.008; *p* = 0.930). Estimated marginal means also indicated similar perceived exertion between formats (3 vs. 3: M = 4.256; SE = 0.267; 95% CI [3.732; 4.779]; 6 vs. 6: M = 4.289; SE = 0.320; 95% CI [3.662; 4.915]; Δ = −0.033; SE = 0.379; *p* = 0.930; 95% CI [−0.777; 0.710]). After applying false discovery rate correction using the Benjamini–Hochberg procedure across the primary GEE model-effect tests, the condition × moment interaction for tension remained statistically significant (q = 0.032). The main effects of moment for state anxiety, depression, anger, fatigue, and vigor also remained statistically supported after correction. In contrast, the condition × moment interaction for anger, the main effect of moment for confusion, and the main effect of condition for enjoyment did not remain statistically robust after FDR adjustment. Accordingly, the unadjusted estimates, including Δ, SE, 95% CI, *p*-values, and Cohen’s d, are presented descriptively for transparency, but these effects should be interpreted cautiously as exploratory findings.

## 4 Discussion

In this repeated-measures study, formal and small-sided volleyball games produced broadly similar acute psychological responses across most outcomes in male youth athletes during the pre-competitive period. Nevertheless, the formats were not psychologically equivalent in all respects. The reduced format was associated with less favorable responses on specific mood dimensions, particularly tension, whereas the findings for anger and enjoyment should be interpreted with caution because they did not remain robust after false discovery rate correction. Overall, the practical magnitude of most observed differences was small, but the pattern of results suggests that game format may be associated with different short-term emotional experiences during training.

State anxiety increased from pre- to post-session in both game formats, indicating that participation in these tasks was associated with greater acute emotional activation rather than with immediate anxiolytic effects. In the present pre-competitive context, this pattern may reflect the intense psychological and physiological engagement required in both small-sided and formal games, including attentional demands, rapid decision-making, and social perception processes (Freitas et al., 2014; Zhao et al., 2024). Although acute increases in emotional activation have sometimes been discussed in relation to performance and emotional learning processes in developing athletes (Jekauc et al., 2021), the present data do not allow us to determine whether this response was functionally beneficial or detrimental. Additionally, greater individual exposure and lower action predictability may intensify situational anxiety, particularly in reduced formats, whereas more stable game structures may attenuate these responses in non-competitive contexts (Clemente, 2025; Halouani et al., 2023). However, because data collection took place during the pre-competitive period, heightened pressure to demonstrate superior performance in selecting starters and reserves may also have contributed to the observed increase in anxiety, regardless of game condition.

Regarding mood, the most consistent and statistically robust condition-related difference was observed for tension, with higher post-session values in the 3 vs. 3 condition than in the 6 vs. 6 condition, yielding a small-to-moderate effect size (*d* = 0.65). Importantly, this interaction remained statistically significant after false discovery rate correction, underscoring the robustness of this finding despite the analysis of multiple psychological outcomes. For anger, a similar post-session pattern was observed; however, the corresponding effect size was small (*d* = 0.40), and the interaction did not remain statistically robust after FDR adjustment. Therefore, this finding should be interpreted cautiously as exploratory. At the same time, the increases in depression, fatigue, and confusion and the decrease in vigor from pre- to post-session, irrespective of condition, are consistent with prior evidence showing that intense training sessions may acutely alter mood profiles in youth athletes (Rotta et al., 2014; Zeng et al., 2023). The higher post-session tension observed in the 3 vs. 3 condition may reflect greater individual exposure and perceived responsibility per action. However, because no objective external-load variables (e.g., ball contacts, jump frequency, action density, or movement demands) or physiological markers (e.g., heart-rate responses or heart-rate variability) were collected, the present study cannot determine whether the observed emotional responses were mediated by greater physical, technical, or cognitive involvement in the reduced format. Consequently, the proposed explanatory mechanisms remain speculative and should be interpreted with caution until verified through multimodal psychophysiological monitoring.

An additional point that warrants consideration is that the overall post-session profile observed in the present study, characterized by increased anxiety, fatigue, depression, and confusion, together with reduced vigor, appears less favorable than the acute mood improvements often described in the classical exercise literature. This apparent discrepancy may reflect important contextual differences between general exercise settings and pre-competitive youth sport environments. Much of the literature reporting acute psychological benefits of exercise is based on recreational, health-oriented, or non-evaluative contexts, whereas the present study was conducted during a pre-competitive period involving trained youth athletes exposed to performance demands and possible evaluative pressure (Jekauc et al., 2021; Putukian and Yeates, 2023). In volleyball, pre-competitive anxiety and emotional fluctuations have also been reported in youth categories, reinforcing the contextual sensitivity of these responses (de Andrade Nogueira et al., 2024). Under these circumstances, acute emotional activation, transient fatigue, and mood disturbance may reflect immediate task stress and contextual demands rather than contradict the broader literature. Therefore, the present findings should be interpreted as context-dependent responses within competitive youth sport rather than as evidence against the general mood-enhancing effects of physical activity.

Taken together, the present findings may be better understood through an integrative framework in which the game format may be associated with differences in the immediate training experience, including perceptual-cognitive demands, action density, individual exposure, and social-evaluative pressure. In reduced formats such as 3 vs. 3, players may be required to engage more frequently in decision-making, assume greater responsibility per action, and remain more consistently involved in the task, all of which may intensify cognitive and emotional strain (Jekauc et al., 2021; Rocha et al., 2020). By contrast, the formal game may distribute demands across a larger number of players and more closely reproduce the organizational structure with which these athletes are familiar in competition (Nascimento et al., 2023; de Andrade Nogueira et al., 2024). Although these mechanisms were not directly assessed, this framework may help explain why the formats were broadly similar across several outcomes, yet showed a statistically robust difference for tension and exploratory, non-robust patterns for anger and enjoyment.

Regarding affective valence, no between-condition difference was observed. Previous studies have suggested that small-sided games may elicit more positive affective responses than more traditional training methods, partly because their playful and decision-rich context can enhance engagement and satisfaction (Clemente, 2025; Zeng et al., 2023). Contextual factors such as verbal encouragement may also influence acute affective responses during game-based training (Clemente, 2025; Selmi et al., 2017). In the present study, however, affective valence remained similar across formats, suggesting that the immediate hedonic tone of the experience did not differ meaningfully between 3 vs. 3 and 6 vs. 6 in this group of youth volleyball athletes.

Enjoyment scores were descriptively more favorable for 6 vs. 6 than for 3 vs. 3. However, this effect did not remain statistically robust after false discovery rate correction and should therefore be interpreted with caution. In this competitive sample, the higher enjoyment observed in the formal game may reflect its closer alignment with the tactical organization and performance demands typically experienced by trained volleyball players (Clemente, 2025; Zeng et al., 2023). From a motivational perspective, this pattern may also be tentatively interpreted in light of self-determination theory, particularly the possibility that enjoyment was influenced by how closely the task matched athletes’ perceived competence and role familiarity. In competitive youth volleyball players, the formal game may have provided a context more consistent with their positional expectations, tactical references, and prior sport experience, thereby favoring a more positive appraisal of the session (Nascimento et al., 2023; Selmi et al., 2020). In this sense, the greater enjoyment observed in the formal format may reflect not only a preference for the official structure but also a closer fit between perceived ability and task demands (Clemente, 2025; Zeng et al., 2023). This interpretation may also help explain why the present findings differ from studies in invasion sports, in which small-sided games often generate greater enjoyment through continuous participation and broader decision-making opportunities (Selmi et al., 2020; Zeng et al., 2023). In volleyball, however, the formal game may be perceived as more meaningful and rewarding because it better reproduces tactical coordination, role specificity, and the emotional dynamics of competition (Nascimento et al., 2023). Nevertheless, because intrinsic motivation and perceived competence were not directly measured, and because the observed effect size was small (*d* = 0.54), this interpretation should be considered cautiously.

The recovery and RPE variables did not differ between conditions. This result suggests that, despite some differences in emotional responses, both game formats imposed a broadly comparable perceptual load on the athletes. The weekly training frequency and non-competitive training context may have facilitated adequate recovery of the prescribed load, consistent with expected adaptations during youth sport development (Freitas et al., 2014; Teixeira et al., 2021). In addition, training intensity during formative stages often allows for technical-tactical correction and controlled progression of effort, which may help maintain recovery and perceived exertion within manageable ranges (Freitas et al., 2014; Varghese et al., 2022).

In summary, the results of this exploratory study indicate that, in youth volleyball, reduced and formal game formats were associated with broadly similar acute psychological responses across most outcomes. Among the analyzed variables, greater post-session tension in the reduced game represented the most consistent and statistically robust finding, remaining significant after false discovery rate correction and reaching a small-to-moderate effect size (*d* = 0.65). Although greater post-session anger and higher enjoyment in the formal game were also observed, these findings did not remain statistically robust after correction for multiple comparisons and should therefore be interpreted cautiously as exploratory. Overall, the findings highlight the importance of integrating psychological responses into training planning and avoiding the assumption that small-sided and formal games are psychologically interchangeable. At the same time, these differences should not be interpreted as responses attributable to the game format alone, because the fixed sequence of conditions does not allow for a clean separation between format-related and time-related influences.

However, some limitations should be acknowledged. First, the sample was relatively small, restricted to male youth athletes, and drawn from a specific competitive context, which limits precision and generalizability. Second, although repeated observations were obtained from the same athletes, the study used a fixed, non-counterbalanced sequence of conditions. This represents a central limitation of the design, as order, learning, habituation, accumulated fatigue, and contextual changes across sessions may confound the observed effects of the condition. Therefore, it is not possible to attribute differences between 3 vs. 3 and 6 vs. 6 exclusively to the game format itself. Third, although the repeated-measures structure was accounted for in the GEE models, session/trial was not included as an explicit fixed effect. Consequently, the analyses do not allow direct examination of temporal trends across repeated exposures, such as adaptation, habituation, or residual order-related influences. In addition, potentially relevant moderating variables such as court position, biological maturation, effective playing time, competitive role within the team (e.g., starters vs. reserves), and training experience were not incorporated into the analytical models. These characteristics may meaningfully influence perceived responsibility, emotional regulation, stress appraisal, and psychological responses during training tasks. Consequently, the present analyses cannot determine whether different athlete profiles respond differently to reduced and formal game formats. Another limitation is that psychological assessments were restricted to pre-session and immediate post-session measurements. Therefore, the study does not provide information regarding the short-term recovery trajectory or temporal persistence of the observed emotional responses. Future studies should incorporate subgroup and moderation analyses to examine whether acute psychological responses vary according to developmental, tactical, and competitive characteristics of youth athletes, and should include additional follow-up measurements, such as 15-min and 30-min post-session assessments, to better characterize the recovery dynamics of anxiety and mood responses.

From an applied perspective, the present findings suggest that coaches may use both formal and small-sided game formats during youth volleyball training, but they should not assume that these structures are psychologically interchangeable. The apparent combination of greater individual involvement and less favorable emotional responses in the reduced format indicates that task design may simultaneously stimulate engagement and increase short-term emotional strain. This has practical relevance because formats chosen to intensify participation or decision-making may also alter how athletes experience the session emotionally, particularly during pre-competitive periods. Conversely, the descriptively higher enjoyment observed in the formal format, although not robust after FDR correction, suggests that perceived familiarity, tactical organization, and role specificity may warrant consideration when the goal is to promote a more positively experienced training environment. Therefore, monitoring short-term psychological responses alongside training content may help coaches better align pedagogical objectives, emotional demands, and athlete readiness.

## 5 Conclusion

In this repeated-measures study, formal and small-sided volleyball games produced broadly similar acute psychological responses in male youth athletes across most assessed outcomes. However, the 3 vs. 3 format was associated with higher post-session tension, with a small-to-moderate effect size (*d* = 0.65), and this was the only condition-related finding that remained statistically robust after false discovery rate correction. Although higher post-session anger in the 3 vs. 3 format and greater enjoyment in the 6 vs. 6 format were also observed, these effects were not statistically robust after correction for multiple comparisons and should therefore be interpreted with caution. State anxiety increased after the sessions regardless of format, and no clear between-condition differences were observed for affective valence, perceived recovery, or perceived exertion. In addition, depression, fatigue, and confusion increased, and vigor decreased from pre- to post-session, irrespective of game format, all with small effect sizes. Taken together, these exploratory findings suggest that reduced and formal volleyball games may be associated with distinct short-term emotional responses during youth volleyball training, although most observed differences were of limited practical magnitude. Because the study was based on a small, nonrandomized sample and relied exclusively on self-reported outcomes, the findings should be interpreted with caution and treated as hypothesis-generating.

## Data Availability

The original contributions presented in the study are included in the article/Supplementary material, further inquiries can be directed to the corresponding author.
