# Correction: Acute psychological responses to formal and small-sided volleyball games in male youth athletes: an exploratory repeated-measures study

**DOI:** 10.3389/fpsyg.2026.2021594

**Published:** 2026-10-19

**Authors:** Marcos Henrique do Nascimento, Alberto Souza de Sá Filho, Vicente Aprigliano, Filipe Manuel Clemente, Carlos Alexandre Vieira, Mário Hebling Campos, Augusto Cezar Rodrigues Rocha, Débora Darck Lopes Costa Arantes, Gibson Moreira Praça, Auro Barreiros Freire, Adam Kawczyński, Matias Noll, Gustavo De Conti Teixeira Costa

**Affiliations:** 1 Faculdade de Educação Física, Universidade Federal de Goiás, Goiânia, Brazil; 2 Universidade Evangélica de Goiás, Anápolis, Brazil; 3 Escuela de Ingeniería de Construcción y Transporte, Pontificia Universidad Católica de Valparaíso, Valparaíso, Chile; 4 Gdansk University of Physical Education and Sport, Gdańsk, Poland; 5 Applied Research Institute (i2A), Sport Physical Activity and Health Research and Innovation Center, Polytechnic University of Coimbra, Coimbra, Portugal; 6 Escola de Educação Física, Fisioterapia e Terapia Ocupacional, Universidade Federal de Minas Gerais, Belo Horizonte, Brazil; 7 Instituto Federal de Minas Gerais, Campus Sabará, Sabará, Brazil; 8 Faculty of Medicine, Wrocław University of Science and Technology, Wrocław, Poland; 9 Instituto Federal de Educação, Ciência e Tecnologia Goiano, Ceres, Brazil

**Keywords:** enjoyment, mood state, small-sided games, state anxiety, volleyball, youth athletes

In the Acknowledgments, an acknowledgments statement was omitted from the article. The following statement should be added: “We want to thank the Universidade Federal de Goiás (UFG), the Fundação de Amparo à Pesquisa do Estado de Goiás (FAPEG), the Instituto Federal de Educação, Ciência e Tecnologia Goiano—Campus Ceres, and the Conselho Nacional de Desenvolvimento Científico e Tecnológico (CNPq) for their support of this research.”

The original version of this article has been updated.

